# Microperforated Compostable Packaging Extends Shelf Life of Ethylene-Treated Banana Fruit

**DOI:** 10.3390/foods11081086

**Published:** 2022-04-09

**Authors:** Victor Rodov, Ron Porat, Amit Sabag, Bettina Kochanek, Haya Friedman

**Affiliations:** Agricultural Research Organization, The Volcani Institute, Rishon LeZion 7505101, Israel; rporat@volcani.agri.gov.il (R.P.); amitsa@volcani.agri.gov.il (A.S.); betina@volcani.agri.gov.il (B.K.); hayafr@volcani.agri.gov.il (H.F.)

**Keywords:** biodegradable packaging, sustainability, banana, postharvest quality, perforation, modified atmosphere, ethylene, oxygen, fermentation volatiles, senescence spotting

## Abstract

Plastic packaging preserves the quality of ethylene-treated bananas by generating a beneficial modified atmosphere (MA). However, petroleum-based plastics cause environmental pollution, due to their slow decomposition. Biodegradable packaging may help resolve this controversy, provided it shows adequate preservation efficacy. In this study, we tested the compostable biodegradable polyester packaging of ethylene-treated bananas in comparison with commercially available petroleum-based plastic alternatives. When compostable packaging was used in a non-perforated form, it caused hypoxic fermentation, manifested as impaired ripening, off-flavor, and excessive softening. Micro-perforation prevented fermentation and allowed MA buildup. Furthermore, no water condensation was observed in the biodegradable packages, due to their somewhat higher water vapor permeability compared to conventional plastics. The fruit weight loss in biodegradable packaging was higher than in polypropylene, but 3–4-fold lower than in open containers. The control of senescence spotting was the major advantage of microperforated biodegradable packaging, combined with the preservation of acceptable fruit firmness and flavor, and low crown rot incidence. Optimal biodegradable packages extended the shelf life of bananas by four days compared with open containers, and by two days compared with the best commercial plastic package tested. Microperforated biodegradable packages combined the advantage of improved sustainability with superior fruit preservation.

## 1. Introduction

Banana is the fourth largest food crop [1] and the most important traded fruit in the world, with an annual global trade of USD 11 billion [2]. Banana is a typical climacteric fruit, whose ripening depends on ethylene action [3]. The fruit has a relatively narrow time window of optimal edible quality between the under-ripe and overripe stages, and special technological interventions are needed in order to ensure that the bananas reach the consumers in the best condition [1]. In particular, ethylene gassing or other treatments, such as ethrel (ethephon) or acetylene applications, are used commercially prior to distribution and marketing, in order to synchronize the fruit ripening, reducing the natural variation in their maturity within a bunch or among bunches [4].

However, ethylene-treated bananas are very perishable and their life typically does not exceed 3–5 days [5]. The appearance of peel brown specs (senescence spotting) marks the onset of banana over-ripening [6]; the spots promptly coalesce and spread over the fruit surface, rendering it unmarketable. At the advanced ripening stage, the bananas are also prone to diseases, such as crown rot and anthracnose [7,8]. Several approaches have been tested, in order to extend the life of ethylene-treated bananas, including keeping them at optimal storage temperature [5,9], applying the inhibitor of ethylene action 1-methylcyclopropene (1-MCP) [5,10], short-term vacuum exposure, and modified atmosphere packaging (MAP) [9,11]. The enclosure of bananas, prior to marketing, in consumer-sized retail packages helps maintain their quality during shelf life and home storage [12].

MAP, containing lower oxygen and higher carbon dioxide levels as compared to regular air, develops inside semipermeable plastic packages, due to the interaction between the respiratory activity of the fresh produce and the diffusion of gases through the packaging material [13]. MAP can help banana preservation at the following two supply chain links: during distant shipment, by maintaining the mature-green fruit state, and during distribution/marketing of the ethylene-treated fruit. Packaging in low-density polyethylene (LDPE) bags can extend the storage life of shipped mature-green bananas by up to 30 days at 14 °C [8]. The modified atmosphere (MA) composition recommended for the marine transportation of bananas is 2–5% for both oxygen and carbon dioxide levels [14]. During distribution and marketing, MAP slows down the aging of ethylene-treated bananas, in particular, by inhibiting senescence spotting. Choehom et al. [15] showed that the latter effect was due to reduced oxygen (partial pressure 5 to 10 kPa), rather than enhanced carbon dioxide accumulation. In another study, MAP with 12% O_2_ and 11% CO_2_ was found to be the most efficient for delaying the ripening of ethylene-treated bananas [11]. At the same time, keeping bananas under hypoxic conditions (below 1% oxygen) results in ripening disorders, such as “green ripe”, when arrested peel color change is combined with pulp softening and off-flavor development [8,16].

While plastic packaging can help preserve perishable commodities, such as bananas, the extensive use of plastics results in environmental pollution, due to the very slow decomposition of conventional petroleum-based polymers [17]. Compostable, biodegradable packaging may improve the sustainability of the fruit and vegetable industry by preserving perishable produce with lower pressure on the environment [18]. Shifting the plastic industry from traditional fossil fuel-derived polymers to plant-based biodegradable materials has been referred to as the second green revolution [19]. However, if greenhouse gas (GHG) emission is taken into account, it appears that the overall environmental impact of biodegradable packaging is only positive if its efficacy in food loss reduction is at least equal to that of conventional plastics [20]. Therefore, the performance of biodegradable films for controlling food spoilage is critical for the realization of their environmental promise. The information on biodegradable packaging performance in fruit and vegetable storage is very scarce, although positive results on the preservation of mature-green bananas in biodegradable ‘Bioflex’ packaging were reported by Abdul-Rahaman and Bishop [21]. The effect of biodegradable packaging on the storage of ethylene-treated bananas has not been investigated previously.

In this study, we tested the efficacy of the compostable bio-based packaging film developed by TIPA Corp. (Israel) for the preservation of fresh ethylene-treated bananas. The performance of the biodegradable film was checked during a simulated supply chain, in comparison with commercial petroleum-based flexible plastic packages and open rigid containers.

## 2. Materials and Methods

### 2.1. Plant Materials

Banana fruit, cv. Cavendish Grande Naine, were purchased from a packinghouse in Jordan Valley, Israel, after the application of a commercial ethylene degreening treatment, and were brought to the Volcani Center afterwards. The fruit were divided into clusters, each containing 6–7 fruit and weighing about 1 kg, at a uniform ripening stage of light green/beginning of yellowing (stage 3). The clusters were distributed into seven packaging treatments, as described below, with one cluster per package. Each treatment included nine packages, with three packages for each evaluation point, and each package/cluster serving as a replication.

### 2.2. Packaging Materials

The compostable bags (dimensions 300 × 400 mm) of 35 µm biodegradable polyester blend film [22] were supplied by TIPA Corp. (Hod HaSharon, Israel). Film micro-perforation was performed using a cold 0.5 mm needle; in agreement with the preliminary results, the micro-perforated packages bore 16 perforations per package, distributed in two rows in the middle part of the bag. Macro-perforation (6 mm) was performed by film punching, with eight perforations per package. The performance of the biodegradable packages was compared with commercial packaging materials of micro- or macro-perforated 35 µm thick cast polypropylene (CPP), designated as Commercial-1 (COM-1), and polyamide-based film (PA), designated as Commercial-2 (COM-2). The control samples were kept in open rigid polyethylene terephthalate (PET) containers.

The transmission rate tests were performed at Israel Plastics & Rubber Center (Ramat Gan, Israel). The oxygen transmission rate (OTR) was determined using a MOCON OX-TRAN 2/22H device, in accordance with the ASTM D-3985 guidelines, in duplicate, with a testing area of 5 cm^2^. The average OTR values were measured as 2019 cm^3^ (m^2^ day)^−1^ for the CPP film and 876 cm^3^ (m^2^ day)^−1^ for the compostable film. The water vapor transmission rate (WVTR) of the CPP film was determined as 8.1 g (m^2^ day)^−1^ using a PERMATRAN-W 3/34G device, in accordance with the ASTM F-1249 guidelines. Since the ASTM method is not suitable for highly permeable films [23], the WVTR of the continuous compostable film was determined at the ARO, according to the modified method suited for testing hydrophilic edible films [24], and was found to be 40.8 g (m^2^ day)^−1^.

The actual transmission rates of the microperforated packages were calculated in agreement with the mathematical model of Fishman et al. [25], based on the relative area of perforations in the total package surface, and the values of oxygen and water vapor diffusion coefficients in the air at 25 °C [26]. Using this approach, the OTR and WVTR values of the microperforated biodegradable packages used in this study were estimated as 40.9 × 10^4^ cm^3^ (m^2^ day)^−1^ and 41.15 g (m^2^ day)^−1^, respectively. In agreement with our previous calculations [27], the perforation resulted in a great 46-fold increase in the film’s oxygen transmission rate and just a negligible change in the permeability towards water vapor. Altogether, the perforations accounted for 97.8% of the oxygen flux through the package, but only 0.85% of the water vapor flux, which mainly passed through the film matrix. For comparison, in CPP film with the same perforation level, the perforations would be responsible for 95.2% of the oxygen transmission and 4.1% of the water vapor exchange.

### 2.3. Storage Regimes

**Figure 1:**
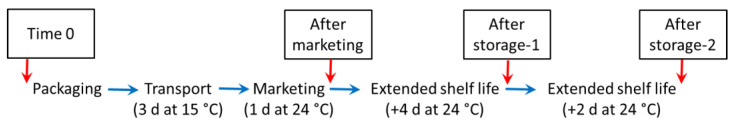
Schematic illustration of the transport, marketing, and storage of bananas. Red arrows indicate the time points for quality evaluations.

The scheme of the simulated transport, marketing, and storage regime of bananas is presented in Figure 1.

Produce quality evaluations were conducted at four time points: (a) at the beginning of the experiment (time zero); (b) after 3 days of distribution at 15 °C + one day of marketing at 24 °C; (c) after distribution and marketing + two additional days at 24 °C; (d) after distribution and marketing + four additional days at 24 °C. The additional days at 24 °C simulated an extended shelf life period or non-refrigerated storage of bananas at the consumer’s home. Three packages from each treatment were taken for quality evaluations at each time point, and each package was considered as one replication.

### 2.4. Quality Evaluation

The headspace oxygen and carbon dioxide concentrations in the packages were measured with an OXYBABY gas analyzer (WITT Gasetechnik GmbH & Co KG, Witten, Germany). The atmosphere samples (ca. 6 mL) were withdrawn from the package by inserting the analyzer’s needle into the headspace through foam rubber sticker seals (WITT Gasetechnik), ensuring the gas tightness of sampling. For the analysis of ethylene and fermentative volatiles (ethanol and acetaldehyde vapours), the headspace samples (ca. 5 mL) were withdrawn from the packages with gastight syringes, through the same rubber seals. The volatiles were analyzed using a Varian 3300 gas chromatograph (Varian, Walnut Creek, CA, USA), equipped with a flame ionization detector (FID), using helium as the carrier gas and authentic external standards for quantification. The acetaldehyde and ethanol vapors were analyzed using a 20% Carbowax 20 M packed column; the column, injector, and detector temperatures were 80, 110, and 180 °C, respectively. A stainless-steel column (length 1.5 m and internal diameter 2.16 mm) packed with HayeSep T (Alltech Associates, Inc., Deerfield, IL, USA) was used for ethylene analysis; the column, injector, and detector temperatures were 80, 50, and 56 °C, respectively. The amount of condensed water in the packages was evaluated visually according to the 4-grade abundance scale, where 0 = none, 1 = slight, 2 = moderate, and 3 = profound.

The fruit weight loss during storage was calculated as a percentage of the initial weight. The crown rot severity was characterized as the incidence of infected clusters out of the three replications. Flavor rating was performed according to the 9-grade hedonic scale, where 1 = very bad and 9 = very good, with a score of 5 as the minimum flavor acceptability threshold. The flavor rating was assigned by a consensus decision of the panel.

The fruit firmness was evaluated using a universal testing machine Inspekt TABLE BLUE 5 kN, desktop model (Hagewald & Peschke, Nossen, Germany). The firmness was measured as compression force (N), causing 3% strain on the fruit, using compression plates that were 30 mm in diameter with a crosshead speed of 5 mm min^−1^.

The color of the bananas was evaluated visually according to the 8-grade scale, where 1 = dark-green, 2 = light green, 3 = breaker, 4 = greenish yellow, 5 = yellow with a green tip, 6 = yellow, 7 = yellow flecked with brown, and 8 = brown. In addition, the appearance of brown speckles or spots on the banana peel surface was evaluated according to the 4-grade scale, where 0 = none, 1 = slight, 2 = moderate, and 3 = profound discoloration. A visual acceptance score was given according to the 5-grade scale, where 1 = very bad, 2 = bad, 3 = moderate, 4 = good, and 5 = excellent. A score of 2.5 was defined as the visual acceptability threshold. In all visual or sensory evaluation tests, the scores were given by three trained panelists.

### 2.5. Statistical Analysis

All evaluations or measurements were performed at least in triplicate. A Microsoft Office Excel spreadsheet was used to calculate means, standard deviations, and standard errors. A one-way analysis of variance (ANOVA) and Tukey’s honestly significant difference (HSD) pairwise comparison tests were performed using the JMP statistical software program, version 7 (SAS Institute Inc., Cary, NC, USA).

## 3. Results

**Figure 2:**
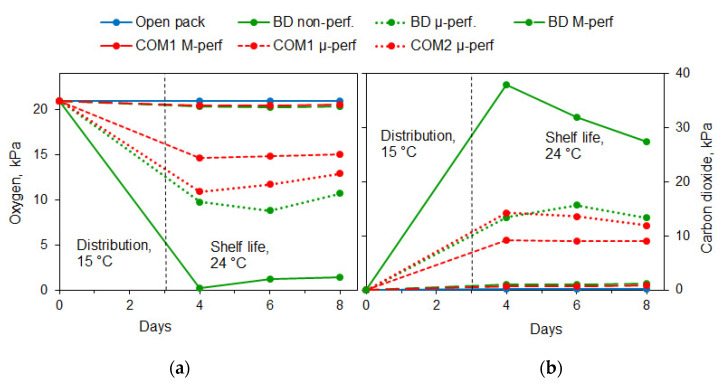
Headspace oxygen (**a**) and carbon dioxide (**b**) levels in banana packages during simulated distribution at 15 °C and shelf life/home storage at 24 °C. Package types: control (Open pack), non-perforated, micro- and macro-perforated biodegradable packages (BD non-perf., BD µ-perf. and BD M-perf., resp.), macro-and micro-perforated commercial CPP packages (COM1 M-perf. and COM1 µ-perf., resp.), and micro-perforated commercial PA-based package (COM2 µ-perf.).

**Figure 3:**
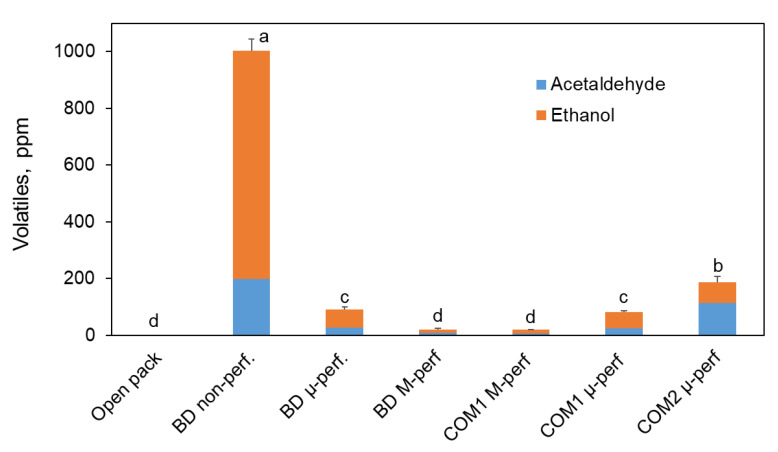
Accumulation of acetaldehyde and ethanol vapors (fermentation volatiles) in the headspace of banana packages after 3 days of simulated distribution at 15 °C and 5 days of shelf life/home storage at 24 °C. Package designations are the same as in Figure 2. Error bars represent standard errors of three replications. Different letters above the bars indicate significant differences at *p* ≤ 0.05 according to Tukey’s HSD test.

Keeping bananas in non-perforated biodegradable packages caused almost complete oxygen depletion (Figure 2a), resulting in anaerobic fermentation, manifested as disproportionately high carbon dioxide accumulation (Figure 2b) and the accumulation of fermentation volatiles, especially ethanol vapor (Figure 3). The atmosphere composition in the macro-perforated packages was very similar to that in the open containers. On the other hand, the micro-perforated packages supported the generation of modified atmospheres with reduced O_2_ and enhanced CO_2_ levels. The steady-state O_2_ and CO_2_ levels established in micro-perforated biodegradable packages were 8–11 and 12–15 kPa, respectively. In commercial micro-perforated packages, the O_2_ levels varied between 12 and 16 kPa, and the CO_2_ levels between 8 and 13 kPa.

**Figure 4:**
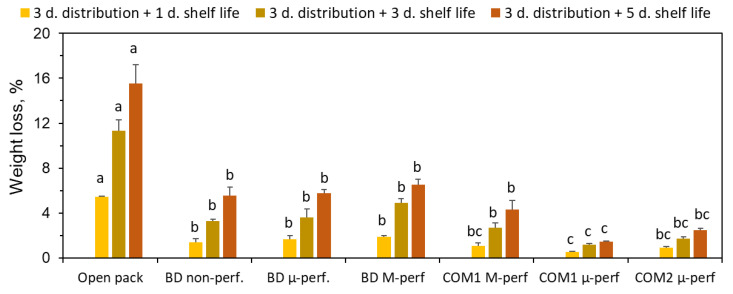
The effect of packaging methods on the weight loss of banana fruit after 3 days of simulated distribution at 15 °C and 1, 3 or 5 days of shelf life/home storage at 24 °C. Package designations are the same as in Figure 2. Error bars represent standard errors of three replications. Within each sampling day, different letters above the bars indicate significant differences between packaging types at *p* ≤ 0.05, according to Tukey’s HSD test.

As expected, by far the highest weight loss was registered in the open control containers. The lowest weight loss was observed in the micro-perforated polypropylene-based commercial packages. Increasing the perforation area or using PA-based packaging material both resulted in a certain increase in weight loss (Figure 4). Even higher weight loss was observed in the packages made of biodegradable film, indicating its relatively high water vapor transmission rate. Interestingly, the degree of perforation had a negligible effect on the fruit’s weight loss, confirming that the film matrix, rather than the perforations, served the major pass for water vapor diffusion. Still, bananas stored in the biodegradable packages lost 3–4 times less weight than in the control.

**Figure 5:**
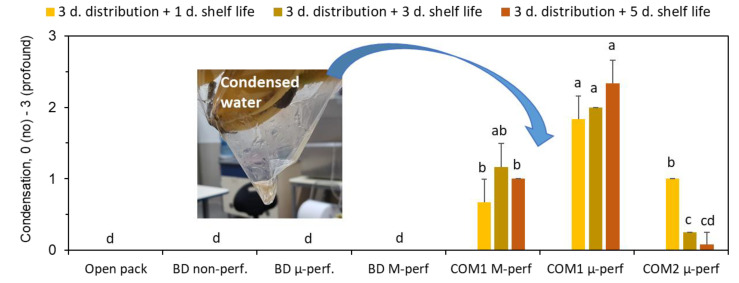
Accumulation of condensed water (visual scores) in banana packages after 3 days of simulated distribution at 15 °C and 1, 3 or 5 days of shelf life/home storage at 24 °C. Package designations are the same as in Figure 2. Error bars represent standard errors of three replications. Different letters above the bars indicate significant differences at *p* ≤ 0.05, according to Tukey’s HSD test. Insert represents a photograph of condensed water accumulated on the bottom of the commercial microperforated CPP-based package.

The biodegradable packages contained no condensed water, while profound condensation was registered in the commercial CPP-based packages (Figure 5). A small amount of condensed water was also observed in the PA-based commercial packages after the storage temperature shift, but it diminished during further shelf life.

Mold development on the cut surfaces of the banana crowns was affected by the packaging material and perforation. After five days of shelf life, moldy cuts were observed in all the clusters kept in macro-perforated packages and in microperforated CPP bags, causing the most profound water condensation. No mold growth occurred in the hypoxic non-perforated biodegradable packages, while the microperforated biodegradable or PA-based bags and open containers supported limited crown rot development in one or two of the three replications.

**Figure 6:**
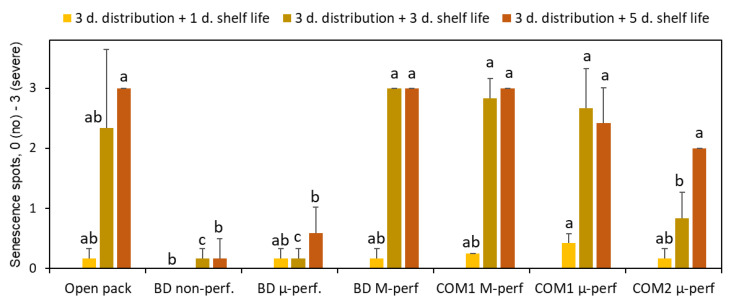
The effect of packaging methods on the severity of banana peel discoloration (senescence spotting) after 3 days of simulated distribution at 15 °C and 1, 3 or 5 days of shelf life/home storage at 24 °C. Package designations are the same as in Figure 2. Error bars represent standard errors of three replications. Within each sampling day, different letters above the bars indicate significant differences between packaging types at *p* ≤ 0.05, according to Tukey’s HSD test.

**Figure 7:**
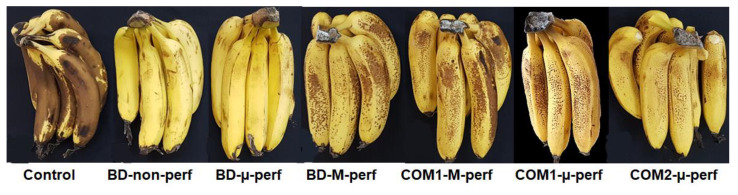
Fruit appearance after three days of simulated distribution at 15 °C and five days of shelf life/home storage at 24 °C. Package designations are the same as in Figure 2.

Storage in non-perforated or micro-perforated biodegradable packages controlled the banana senescence spotting (Figure 6), initially appearing as brown speckles that further coalesced into big spots (Figure 7). The commercial PA-based packages also inhibited the speckle appearance, especially during the first three days of shelf life/home storage.

**Figure 8:**
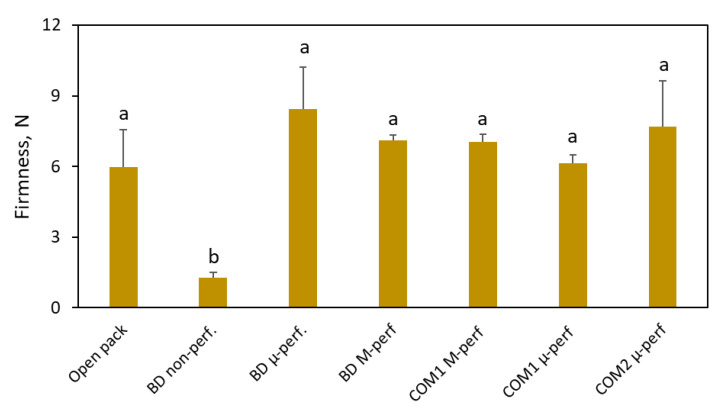
The effect of packaging on the firmness of bananas after 3 days of simulated distribution at 15 °C and 5 days of shelf life/home storage at 24 °C. Package designations are the same as in Figure 2. Error bars represent standard errors of three replications. Different letters above the bars indicate significant differences at *p* ≤ 0.05, according to Tukey’s HSD test.

Although the fruit kept in non-perforated packages had an acceptable visual appearance throughout the trial period, showing almost no senescence spotting and crown rots, it suffered acute physiological hypoxic damage, manifested as severe softening (Figure 8) and prohibitive off flavor (data not shown). No substantial taste and firmness difference was detected among the other packaging treatments.

**Figure 9:**
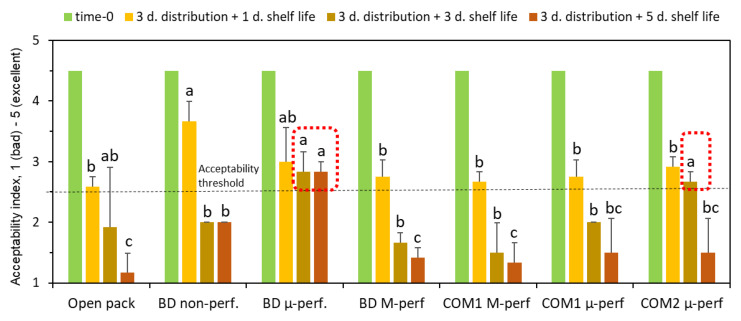
The effect of packaging methods on general acceptability of bananas after 3 days of simulated distribution at 15 °C and 1, 3 or 5 days of shelf life/home storage at 24 °C. Package designations are the same as in Figure 2. Error bars represent standard errors of three replications. Within each sampling day, different letters above the bars indicate significant differences between packaging types at *p* ≤ 0.05, according to Tukey’s HSD test. Red dotted frames designate packaging treatments that maintain fruit quality above the acceptability threshold after the extended shelf life of 3 and 5 days.

After simulated distribution and one day of shelf life, the fruit’s overall quality scores were acceptable in all the packages, including the open control (Figure 9). However, after an additional two days at 24 °C, the acceptable quality was maintained in only two micro-perforated package types, those of the biodegradable film and of the commercial PA-based package. After two more days, the micro-perforated biodegradable package was the only package that maintained the fruit quality above the acceptability threshold. Thus, the shelf life of bananas kept in optimal biodegradable packages was extended by four days, as compared with fruit stored in open containers, and by two days as compared with the best commercial package tested.

## 4. Discussion

To the best of our knowledge, this work is the first report demonstrating the shelf life extension of ethylene-treated bananas using compostable packaging material. Importantly, this study has shown that, in addition to the advantage of improved sustainability, the bio-based biodegradable polyester film has potential to serve as an excellent packaging material for fresh fruits and vegetables, even outperforming popular petroleum-based plastics, such as polypropylene. These findings corroborate our previous results with other types of fresh produce, e.g., cucumbers [28] and bell peppers [29]. Similar conclusions were made by Mistriotis et al. [30], who investigated the performance of polylactic acid (PLA) as a packaging material for fruits and vegetables. The potential superiority of the bio-based films is due to the relatively high water vapor permeability of their polymeric matrix, reducing the chances of water condensation in the package, at the cost of a somewhat higher weight loss of the produce, in agreement with modified humidity packaging principles [27,31].

At the same time, the performance of bio-based films often needs improvement, in order to reach adequate functionality to meet the requirements of the products packaged [32]. In particular, in addition to high water vapor permeability, biodegradable films typically have high oxygen barrier properties [33]. These properties, desirable for keeping non-respiring foods, are inadequate for the preservation of fruits and vegetables. Therefore, biodegradable films often demand adjustment of their barrier properties towards the requirements of fresh respiring produce [30,34]. Without such adjustment, sealing respiring produce in bio-based films can result in hypoxic damage, rendering the produce inedible, due to off-flavor development and impaired ripening, as observed in our trials with non-perforated biodegradable film.

Micro-perforation is the most efficient way of adjusting the O_2_/CO_2_ permeability of packaging materials. Furthermore, the balance between the degree of perforation and the produce load determines the MA composition, and, therefore, greatly affects the package performance. For example, the inhibition of senescence spotting in bananas was observed at oxygen levels of 5 to 10% (i.e., partial pressure of 5 to 10 kPa), but not at 15% O_2_ [15]. Therefore, the lower efficacy of commercial micro-perforated packages in the maintenance of banana color might be related to a somewhat higher oxygen level (12–15 kPa) in the internal atmospheres of these packages, as compared to 10 kPa in the biodegradable packages. In a separate trial, we observed that this advantage of the micro-perforated biodegradable packages in controlling the banana senescence spotting was canceled by increasing their perforation level (data not shown). Interestingly, similar to the control of senescence spotting in bananas, optimal MA was shown to inhibit the melanosis in shrimps, having the same biochemical mechanism, i.e., the enzymatic oxidation of phenolic compounds, driven by polyphenol oxidase (PPO) [35]. Mathematical modeling can be an efficient instrument for optimizing the package perforation level [36].

## 5. Conclusions

- Microperforated biodegradable packaging allowed the buildup of beneficial MA, preventing hypoxic fermentation and improving fruit preservation.
- The biodegradable packages had somewhat higher water vapor permeability, as compared to conventional plastics, and caused no water condensation.
- The fruit weight loss in biodegradable packaging was higher than in conventional plastic bags, but still 3–4-fold lower than in open containers.
- The control of senescence spotting was the major advantage of microperforated biodegradable packaging, combined with the preservation of acceptable fruit firmness and flavor, and low crown rot incidence.
- The shelf life of bananas in optimal biodegradable packages was extended by four days compared with open containers, and by two days compared with the best commercial plastic package tested.
- Optimally perforated compostable packages combine the advantage of sustainability with beneficial modified humidity and modified atmosphere generation. Keeping ethylene-treated bananas in such packages reduces food losses, with minimal pressure on the environment.

## Data Availability

The data presented in this study are available on request from the corresponding author.
